# Polypharmacy to Clozapine Monotherapy in Treatment-Resistant Schizophrenia: A Case Report and Review of the Literature

**DOI:** 10.7759/cureus.63871

**Published:** 2024-07-04

**Authors:** Syed Ali Bokhari, Lubna Lutfi, Muhanad Elnoor, Beenish Mujahid, Abdelaziz Osman

**Affiliations:** 1 Psychiatry, Al Amal Psychiatric Hospital, Emirates Health Services, Dubai, ARE; 2 Psychiatry and Behavioral Sciences, Al Amal Psychiatric Hospital, Emirates Health Services, Dubai, ARE

**Keywords:** schizophrenia, antipsychotics, psychotropic polypharmacy, clozapine, treatment resistant schizophrenia

## Abstract

This case report discusses a 25-year-old Middle Eastern female with a 14-year history of schizophrenia, managed as an inpatient for nearly eight years. Initially referred to a psychiatrist at age 12, with one-year-long concerns about preoccupation with the idea of having a serious illness, depressed mood, decreased appetite, social withdrawal, and aggression, she underwent multiple admissions, various medication combinations, and electroconvulsive therapy but remained resistant to treatment until clozapine monotherapy was initiated in 2023.

After starting clozapine, improvements were noted in speech, communication, and eye contact, though negative symptoms and bouts of aggression persisted. This case highlights the efficacy of clozapine monotherapy in managing treatment-resistant schizophrenia after years of ineffective polypharmacy treatment.

The importance of clozapine in treating treatment-resistant schizophrenia cannot be understated. Despite its efficacy, clozapine is often underutilised globally due to concerns about adverse effects and the need for blood monitoring, leading to the overuse of antipsychotic polypharmacy. This polypharmacy is associated with higher adverse event rates, increased costs, and uncertain long-term safety.

This case report demonstrates the successful management of treatment-resistant schizophrenia with clozapine monotherapy. The patient’s significant improvement supports the need to prioritise clozapine, highlighting its benefits over polypharmacy and advocating for its broader use to enhance patient outcomes.

## Introduction

Schizophrenia is a severe chronic psychiatric disorder characterized by disturbances in thought, perception, and behaviour, involving a range of cognitive, behavioural, and emotional symptoms. Diagnosis requires recognition of the constellation of symptoms negatively impacting personal, social, or occupational functioning.

The Diagnostic and Statistical Manual of Mental Disorders, Fifth Edition, Text Revision (DSM-5-TR) [1] outlines the criteria for diagnosing schizophrenia. The key criteria are the presence of two or more of the following symptoms, each present for a significant portion of time during a one-month period (or less if successfully treated), with at least one being (1), (2), or (3): (1) delusions, (2) hallucinations, (3) disorganised speech, (4) grossly disorganised or catatonic behaviour, and (5) negative symptoms (i.e., diminished emotional expression or avolition).

There is a markedly lower level of functioning in one or more major areas, such as work, interpersonal relations, or self-care, for a significant portion of the time, prior to the onset of the disturbance. Continuous signs of the disturbance persist for at least six months. This six-month period must include at least one month of symptoms (or less if successfully treated) that meet Criterion A (i.e., active-phase symptoms) and may include periods of prodromal or residual symptoms. During these prodromal or residual periods, the signs of the disturbance may be manifested by only negative symptoms or by two or more symptoms listed in Criterion A present in an attenuated form (e.g., odd beliefs, unusual perceptual experiences).

Antipsychotics are the mainstay of the treatment of schizophrenia. They are classified into first-generation and second-generation antipsychotics (FGA/SGA). Typically, treatment is started with one agent at the lowest therapeutic dose, and according to the patient's response upon proper assessment, the medication can be titrated upwards as needed. If sufficient time passes while the patient is on an optimal dose and the response is still poor, switching to another agent is recommended. If the same occurs with a second agent, then augmenting with a second antipsychotic agent is advisable.

In cases of treatment-resistant schizophrenia, the use of clozapine, an atypical antipsychotic, is usually necessary. It is of utmost importance to monitor the patient’s response and to be on the lookout for adverse effects that can range from sedation, hypersalivation, and weight gain to seizures, constipation, and agranulocytosis [2].

Although the resistant symptoms may be negative or cognitive, the resistance of positive symptoms is generally one of the defining features of treatment resistance [3].

The median lifetime prevalence of schizophrenia is approximately four per 1000 persons [4]. The typical age of onset is between the mid-teens and mid-30s, with the peak age of onset of the first psychotic episode being in the early to mid-20s for males and late-20s for females.

## Case presentation

A 25-year-old Middle Eastern female with no medical co-morbidities had suffered from schizophrenia for 14 years and had been managed as an inpatient for nearly eight years.

The patient was first referred to a psychiatrist by a family physician in November 2009, when she was 12 years old, with a one-year history of the idea of having cancer, depressed mood, decreased appetite, lack of desire to study or attend her classes at school, social withdrawal, and physical aggression towards her sister and classmates. Her family history was insignificant for mental illness but positive for cancer in her mother. The school reported that the patient was attending special needs classes and had requested evaluation for autism or learning disability.

She was started on medications and began following as an outpatient. Her family noticed improvement when she was compliant with the medications. During her visits, the patient reported auditory hallucinations and delusions of reference towards her sisters, leading to physical aggression towards them. Her family also noted negative symptoms and poor communication.

Between 2012 and 2016, she had multiple admissions but never returned to baseline. In April 2016, she was admitted for trying to jump out of a moving car, and this admission lasted until 2023. Different medication combinations were tried and adjusted according to the response, as shown in Table 1. She also received six sessions of electroconvulsive therapy (ECT) in 2017. Despite treatment, she remained symptomatic with hallucinatory behaviour, social withdrawal, poverty of speech, psychomotor retardation, poor self-hygiene, difficulty tending to activities of daily living, and bouts of agitation and aggression, which required frequent rapid tranquilisation and a short course of regular benzodiazepines.

**Table 1:** Psychotropic medications the patient received each year during her hospital course. qAM (quaque ante meridiem): every morning qPM (quaque post meridiem): every afternoon or evening q8hr: every 8 hours q12hr: every 12 hours q24hr: every 24 hours

Year	Psychotropic medications
2016	Sertindole 20 mg q24hr; Olanzapine 20 mg q24hr; Promethazine 50 mg qPM; Procyclidine 5 mg q8hr; Haloperidol 5 mg q8hr; Risperidone Consta injection 50 mg IM; Sodium Valproate Chrono 500 mg q12hr; Lamotrigine 50 mg q12hr; Quetiapine 200 mg q12hr; Carbamazepine 200 mg q12hr; Trifluoperazine 5 mg q12hr; Benzhexol 2 mg q12hr; Gabapentin 300 mg q12hr; Ziprasidone 80 mg q12hr
2017	Gabapentin 400 mg q12hr; Sertindole 20 mg q24hr; Trifluoperazine 10 mg q24hr; Benzhexol 5 mg q8hr; Sodium Valproate Chrono 500 mg q12hr; Olanzapine 10 mg q12hr
2018	Triflupenzine 10 mg q24hr; Sodium Valproate Chrono 500 mg q12hr; Benzhexol 5 mg q12hr; Olanzapine 10 mg q12hr; Promethazine 50 mg qPM
2019	Clozapine 450 mg q24hr; Benzhexol 5 mg q12hr; Promethazine 50 mg qPM; Bisoprolol 2.5 mg qAM; Lorazepam 2 mg q12hr; Lithium 200 mg q12hr; Haloperidol 5 mg q8hr
2020	Clozapine 100 mg qAM + 150 mg qPM; Lithium 200 mg q12hr; Haloperidol 5 mg q8hr; Lorazepam 2 mg q12hr; Promethazine 50 mg qPM; Bisoprolol 2.5 mg qAM; Benzhexol 5 mg q24hr
2021	Clozapine 100 mg qAM + 150 mg qPM; Lithium 200 mg q12hr; Promethazine 50 mg qPM; Bisoprolol 2.5 mg qAM
2022	Clozapine 150 mg q12hr; Lithium 200 mg q12hr; Promethazine 50 mg qPM; Bisoprolol 2.5 mg qAM; Olanzapine 5 mg q24hr
2023	Clozapine 100 mg qAM + 350 mg qPM; Fluoxetine 20 mg q24hr; Sodium Valproate 250 mg qAM + 500 mg qPM

She had fluctuations in response to medications, as there were times when she would cooperate by speaking more spontaneously and verbalising her needs, maintaining eye contact albeit briefly, with less associated hallucinatory behaviour. Aggression and agitation were notably worse during the peri-menstrual period and often required physical as well as chemical restraint. Throughout her hospital stay, her family contacted her and came to visit occasionally, but she did not always agree to see them.

In September 2018, she had a trial of clozapine monotherapy, slowly titrated up to 350 mg daily, which showed minimal improvement in speech, communication, and eye contact and she denied delusions or any perceptual abnormalities. Nevertheless, she continued to have negative symptoms, blunted affect, anhedonia, social withdrawal, bouts of unprovoked verbal and physical aggression, and tendencies to abscond from the ward.

Clozapine was stopped in October 2019 due to decreased absolute neutrophil count and total white blood cell count, respectively reaching 1.12 x 10^3^/mcL (reference range: 2.00-7.00 x 10^3^/mcL) and 3.72 x 10^3^/mcL (reference range: 4.00-11.00 x 10^3^/mcL). She was instead started on lithium 200 mg q12hr and lorazepam 2 mg q12hr, with her blood counts slowly normalising. Clozapine was gradually reintroduced at a lower dose in November 2019.

In early 2023, the patient’s care was transferred to a different senior psychiatrist within the facility, who decided to try the monotherapy approach using clozapine again. At that point, the patient was on clozapine 150 mg q12hr, lithium 200 mg q12hr, olanzapine 5 mg q24hr, and promethazine 50 mg qPM. During this time, the patient was always seen sitting in the same seat, refusing to come to the interview room, rarely interacting with co-patients, and having minimal verbal output whenever interviewed. Blunted affect, poor eye contact, and involuntary orofacial movements were noted.

All psychotropic medications were tapered down, while clozapine was decided to be the primary agent, gently titrated upwards to 450 mg daily in divided doses. A brain MRI without contrast revealed a normal study.

Shortly after optimising clozapine, the patient started showing improvements in terms of taking care of her personal hygiene and activities of daily living, though aggression persisted during menses. Despite ongoing hallucinatory behaviour and minimal verbal output, she was able to articulate herself and answer questions when spoken to, although echolalia was noted at times. She became more cooperative inside the ward and followed ward routines and instructions given by nurses.

She also agreed to be seen in the interview room rather than inside the ward. An OBGYN referral ruled out gynaecological causes that could have been causing pain and aggression. Also, to exclude pre-menstrual syndrome, she was started on fluoxetine 20 mg. Ultrasound imaging for both the abdomen and pelvis revealed no abnormalities, and as she was cleared from the OBGYN side, fluoxetine was also stopped. She was started on sodium valproate 750 mg in divided doses for possible seizure activity, which helped reduce aggression; however, an EEG might be necessary for further evaluation. She was also started on regular fleet enemas for constipation, further decreasing aggression.

Her family, informed of her improving condition, visited her and observed marked improvement that they had not seen over the past seven years. Psychoeducation was provided to them on her condition, the need for close monitoring, and adherence to medications to maintain the current state and to bring her back to the hospital in case of any agitation or difficulty managing her at home. They were also educated on the possibility of her prolonged hospitalisation and long-term early-onset schizophrenia being causative of her poor cognition in general and her poor social skills.

To ease the transition home, the team suggested gradually increasing home visits, starting at a few hours, then overnight, then a few days until they were comfortable keeping her at home full time. She was discharged in late 2023.

A month after discharge from the inpatient unit, the patient was followed up at home by the Community Psychiatry team and was doing very well, with no complaints of aggression or irritability reported, although negative symptoms persisted. Overall, she was stable and the family was happy with her condition.

## Discussion

We searched for studies comparing antipsychotic monotherapy versus antipsychotic polypharmacy in schizophrenia in the online database PubMed. We also searched for studies on the effectiveness of clozapine monotherapy in treatment-resistant schizophrenia (TRS). Treatment-resistant schizophrenia (TRS) is characterised by the inability to treat persistent, primarily positive symptoms of schizophrenia with antipsychotics after two or more trials, even with appropriate dosage, duration, and verified compliance [5, 6]. The literature reports that up to 30-34% of patients diagnosed with schizophrenia may later develop treatment-resistant schizophrenia, although this figure is quite variable [7-11].

As the only evidence-based treatment for resistant schizophrenia, clozapine is an extraordinary antipsychotic drug, with 60-70% of patients responding to treatment [12-14]. There are at least 15 clinical practice guidelines supporting it, and the evidence indicates that it is the only first-line treatment for TRS that works [15-36]. Clozapine has a far higher chance of success when administered early than any other drug, but it has not been utilised much in clinical practice, in either the Middle East or the West [37-39]. Rather, the more typical approach to treating TRS is antipsychotic polypharmacy (APP), also referred to as antipsychotic combination prescription [40].

This could be due to concerns about the adverse effects and the inconvenience of clozapine therapeutic blood monitoring. This implies that many people who would benefit greatly from clozapine during their illness do not receive a prescription for it in a timely manner [41, 42]. Treatment-resistant schizophrenia patients are frequently administered very hazardous, non-evidence-based antipsychotic polypharmacy at high dosages [43]. When clozapine is prescribed, the disease significantly transforms, with the patient's overall functioning and psychotic symptoms improving quickly.

Inappropriate prescribing practices for schizophrenia include underusing clozapine and prescribing APPs. Ideally, one should consider using APP only after all other options for monotherapy have been exhausted. That is not usually the case, though. According to some research, the prevalence of APP use in the US ranges from 10% to 30%, with a range of 7% to almost 50% [44-47]. Among Asian countries, the reported prevalence of APP among hospitalised patients, particularly with schizophrenia, ranges from 20% to 66% [48-50].

Though evidence-based guidelines clearly state that APP should be explored only after many unsuccessful monotherapy treatments, including the use of clozapine, recent trends indicate a gradual move towards APP in the same treatment context [6, 22, 51]. According to a national survey conducted in China, one in three people diagnosed with schizophrenia were using APP. Patients on APP and their families reported greater antipsychotic dosages, more adverse effects, earlier age of onset, and less satisfaction with therapy [51].

A new Japanese study also found that the frequency of adverse drug events (ADEs) such as drowsiness, hyperprolactinemia, hypersalivation, hyperlipidaemia, cognitive impairment, and Parkinsonian symptoms increased with APP compared to monotherapy [52]. While there is little data to support the efficacy of APP, it has been observed that continuous use of the medication has generated questions regarding its long-term safety, mortality, and increasing cost [53-57].

This report's primary goals are to reinforce the value of antipsychotic monotherapy in the treatment of schizophrenia, particularly TRS, and to discourage the overuse of APP in cases of poor response, especially in the absence of a previous clozapine trial. This patient did not achieve remission on subtherapeutic APP dosages. The most effective treatment for TRS has been shown to be clozapine monotherapy, as it was in our instance [10, 32-34, 57].

Clozapine has been shown to be the most effective antipsychotic for treating negative symptoms of schizophrenia, in addition to having the lowest risk of causing extrapyramidal symptoms (EPS). It is still unclear, though, what biological mechanism underlies its greater effectiveness [2, 33, 58]. The best response is obtained by switching to clozapine, even in cases of first-episode schizophrenia [60].

Long-acting injections (LAIs) and clozapine were proven to be the most effective combination in preventing future relapses of schizophrenia [61]. Following seven years of admission to the psychiatric ward, our patient's condition significantly improved and she was eventually discharged with the help of an ideal dose of a single antipsychotic, clozapine.

The limitations of our case are that several variables affected our ability to determine whether or not our patient's current state is her baseline. For example, we do not have an IQ test from years ago to which we can compare our patient's current IQ. Additionally, our patient's early onset of presentation and early diagnosis are risk factors for a worse prognosis. Lastly, extended hospital stays also contribute to poor social skills.

## Conclusions

This case report highlights a 25-year-old female with treatment-resistant schizophrenia, who was commenced on clozapine after being on polypharmacy for years. Our patient is an example of our treatment hypothesis, coinciding with many other reported cases that improved significantly on clozapine monotherapy.
